# Sulfadoxine/Pyrimethamine Intermittent Preventive Treatment for Malaria during Pregnancy

**DOI:** 10.3201/eid1611.101064

**Published:** 2010-11

**Authors:** Philippe Deloron, Gwladys Bertin, Valérie Briand, Achille Massougbodji, Michel Cot

**Affiliations:** Author affiliations: Institut de Recherche pour le Développement, Paris, France (P. Deloron, G. Bertin, V. Briand, M. Cot);; Université Paris Descartes, Paris (P. Deloron, G. Bertin, V. Briand, M. Cot);; Faculté des Sciences de la Santé, Cotonou, Benin (A. Massougbodji)

**Keywords:** Malaria, pregnancy, prevention, sulfadoxine/pyrimethamine, intermittent preventive treatment in pregnancy, parasites, CME, synopsis

## Abstract

Adequate methods for monitoring this treatment are needed.

## MEDSCAPE CME

Medscape, LLC is pleased to provide online continuing medical education (CME) for this journal article, allowing clinicians the opportunity to earn CME credit. This activity has been planned and implemented in accordance with the Essential Areas and policies of the Accreditation Council for Continuing Medical Education through the joint sponsorship of Medscape, LLC and Emerging Infectious Diseases. Medscape, LLC is accredited by the ACCME to provide continuing medical education for physicians. Medscape, LLC designates this educational activity for a maximum of 0.5 *AMA PRA Category 1 Credits*™. Physicians should only claim credit commensurate with the extent of their participation in the activity. All other clinicians completing this activity will be issued a certificate of participation. To participate in this journal CME activity: (1) review the learning objectives and author disclosures; (2) study the education content; (3) take the post-test and/or complete the evaluation at www.medscapecme.com/journal/eid; (4) view/print certificate.

## Learning Objectives

Upon completion of this activity, participants will be able to:

- Identify pregnant women who are most susceptible to pregnancy-associated malaria and the current recommendations for intermittent preventive treatment in pregnancy for malaria prophylaxis
- Examine adverse events resulting from malaria during pregnancy and the efficacy of varying chemoprophylactic regimens in prevention.

## Editor

**Thomas J. Gryczan, MS,** Technical Writer/Editor, *Emerging Infectious Diseases. Disclosure: Thomas J. Gryczan, MS, has disclosed no relevant financial relationships.*

## CME AUTHOR

**Desiree Lie, MD, MSEd**, Clinical Professor; Director of Research and Faculty Development, Department of Family Medicine, University of California, Irvine at Orange. *Disclosure: Désirée Lie, MD, MSEd, has disclosed the following relevant financial relationship: served as a nonproduct speaker for “Topics in Health” for Merck Speaker Services.*

## AUTHORS

*Disclosures:* ***Philippe Deloron, MD, PhD; Gwladys Bertin, MSc; Valérie Briand, MD, PhD, MPH; Achille Massougbodji, MD;*** *and* ***Michel Cot, MD, PhD,*** *have disclosed no relevant financial relationships.*
