# Supplementary material for: Sulfadoxine/Pyrimethamine Intermittent Preventive Treatment for Malaria during Pregnancy
Source: Emerg Infect Dis. 2010 Nov;16(11):1666–70. doi: 10.3201/eid1611.101064 (PMC3294539; doi:10.3201/eid1611.101064)
Supplement: Related texts

# Review article: Sulfadoxine/Pyrimethamine Intermittent Preventive Treatment for Malaria during Pregnancy

**DOI:** 10.3201/eid1611.101064

**Authors:** Philippe Deloron, Gwladys Bertin, Valérie Briand, Achille Massougbodji, Michel Cot

**Affiliations:** Author affiliations: Institut de Recherche pour le Développement, Paris, France (P. Deloron, G. Bertin, V. Briand, M. Cot);; Université Paris Descartes, Paris (P. Deloron, G. Bertin, V. Briand, M. Cot);; Faculté des Sciences de la Santé, Cotonou, Benin (A. Massougbodji)

## Abstract

For monitoring efficacy of sulfadoxine/pyrimethamine intermittent preventive treatment for malaria during pregnancy, data obtained from studies of children seemed inadequate. High prevalence of triple and quadruple mutants in the dihydropteroate synthase and dihydrofolate reductase genes of *Plasmodium falciparum* parasites contrasts with the efficacy of sulfadoxine/pyrimethamine in reducing low birthweights and placental infection rates. In light of this discrepancy, emphasis on using molecular markers for monitoring efficacy of intermittent preventive treatment during pregnancy appears questionable. The World Health Organization recently proposed conducting in vivo studies in pregnant women to evaluate molecular markers for detecting resistance precociously. Other possible alternative strategies are considered.

Malaria during pregnancy is a major cause of anemia and maternal death and one of the main causes of low birthweight (*1*,*2*). Consequently, the World Health Organization (WHO) recommends protection for women during pregnancy. Until recently, prevention consisted of weekly chemoprophylaxis with either chloroquine or sulfadoxine/pyrimethamine. Because of poor patient compliance with prophylaxis and increasing resistance of parasite strains to chloroquine, administration of intermittent preventive treatment in pregnancy (IPTp) with sulfadoxine/pyrimethamine is now recommended for all pregnant women living in areas with stable malaria transmission (*3*). Sulfadoxine/pyrimethamine is given during antenatal visits at curative doses (1,500 mg sulfadoxine and 75 mg pyrimethamine; i.e., 3× the prophylactic dosage previously used) at least twice during pregnancy, once at the second trimester and once at least 1 month after the first treatment.

IPTp with sulfadoxine/pyrimethamine has proven efficacious in reducing the incidence of pregnancy-associated malaria (*4**,**5*) and is currently part of the national malaria prevention program in most countries in Africa. However, resistance to sulfadoxine/pyrimethamine is increasing in Africa (*6**,**7*). In many countries, sulfadoxine/pyrimethamine now demonstrates inadequate therapeutic efficacy in children <5 years of age (*8**–**10*) and is no longer the drug of choice for treatment, having been replaced by artemisinin combination therapy, according to WHO guidelines. Thus, this drug will soon be compromised, and an urgent need exists to assess alternative drug regimens for IPTp.

## Monitoring Drug Efficacy during IPTp with Sulfadoxine/Pyrimethamine

WHO has recently stressed the inadequacy of sulfadoxine/pyrimethamine efficacy data obtained from studies of children <5 years of age with symptomatic malaria as a reliable indicator for pregnant women (*11*). Antimalarial immunity and pregnancy-specific differences in pharmacokinetics explain that in vivo data obtained for these children cannot be extrapolated to adult women (*12**,**13*). Therapeutic efficacy of sulfadoxine/pyrimethamine in children with clinical *Plasmodium falciparum* malaria largely underestimates its efficacy during IPTp because sulfadoxine/pyrimethamine efficacy in pregnant women may likely depend on their previous immunity. Furthermore, primigravidae, who are the most vulnerable to the effects of pregnancy-associated malaria, are also the least protected among pregnant women who are given sulfadoxine/pyrimethamine in areas where resistance is increasing (*13*).

In Tanzania, 28 days after treatment with sulfadoxine/pyrimethamine, the rate of treatment failure was 16% in pregnant women and 80% in children <5 years of age 2 years earlier (*14*). A recent systematic review indicated that 2 doses of IPTp with sulfadoxine/pyrimethamine retained activity to reduce placental malaria and low birthweight in areas with 19%–26% in vivo resistance in children (*5*). Also, the proportional reduction of peripheral parasitemia at delivery compared with that at enrollment with 2 doses of IPTp with sulfadoxine/pyrimethamine remained >60%, even at in vivo resistance rates <39%. In southern Benin, where the in vivo resistance rate to sulfadoxine/pyrimethamine reached 72% in children <5 years of age at day 28 (*9*), IPTp with sulfadoxine/pyrimethamine was still able to reduce the rate of low birthweight by 40% and the proportion of placental infection by 75% compared with the efficacy of chloroquine prophylaxis administered the previous year (*15*). However, a recent study in an area of high drug resistance in Tanzania demonstrated no clinical benefit of IPTp with sulfadoxine/pyrimethamine, in addition to a worse outcome (*16*).

An additional rationale for not extrapolating sulfadoxine/pyrimethamine efficacy data obtained in studies of young children to the efficacy of IPTp is that the primary outcome of interest differs. In children, the main outcome of the in vivo test is parasite clearance. Although parasite clearance is always highly desirable, the main rationale for administering IPTp is to avoid birthweight reduction as a consequence of massive placenta infection. How IPTp achieves such results is unknown. However, parasite clearance may not be required. A high reduction in parasite load in blood is likely to be paralleled in the placenta and may restore transplacental exchanges.

Overall, these findings have led the WHO technical report group to recommend that the protective efficacy of sulfadoxine/pyrimethamine be evaluated in asymptomatic pregnant women instead of in children, in parallel with constant monitoring of the effectiveness of IPTp with sulfadoxine/pyrimethamine at sentinel sites. Another priority identified by the WHO technical group is urgent evaluation of the prevalence of molecular markers associated with drug resistance as a surrogate to the protective efficacy of IPTp (*11*).

## Usefulness of Molecular Methods

The WHO technical report group recommended genotyping of *Plasmodium* spp. dihydrofolate reductase (*dhfr*) and dihydropteroate synthase (*dhps*) genes, a method commonly used in molecular epidemiology, to monitor the protective efficacy of sulfadoxine/pyrimethamine. Numerous molecular epidemiologic studies showed that resistance to pyrimethamine is associated with the acquisition of mutations in *dhfr*; the most common mutations related to pyrimethamine resistance are Ser108Asn, Asn51Ile, Cys59Arg, and Ile164Leu (*17**,**18*). Similarly, resistance to sulfadoxine is associated with 3 mutations in *dhps*: Ala437Gly, Ser436Phe, and Lys540Glu (*19**,**20*). Each mutation leads to a decrease in sensitivity to pyrimethamine (*dhfr* gene) and sulfadoxine (*dhps* gene).

Molecular markers are useful for tracking the emergence and spread of drug resistance where resistance is low or moderate. However, even for markers with virtually absolute correlations between genotype and in vitro phenotype (such as those for sulfadoxine/pyrimethamine), other factors (including acquired immunity and pharmacokinetic parameters) contribute to clearance of drug-resistant parasites, thus explaining the poor correlation with in vivo efficacy.

Djimdé et al. proposed a model accounting for immunity by controlling for age to predict treatment failure rates (*21*). In this model, the genotype-failure index (ratio of prevalence of resistant genotypes to rate of treatment failure in a population) was proposed for mapping resistance by using molecular methods. However, the genotype-failure index model is of particular interest where resistance is still low to moderate (*22*). Conversely, the predictive value of the model is limited when the prevalence of the marker is >80%, approaching fixation in the population (*23*), defined by a mutation being present without drug pressure and recovered in subsequent parasite generations. Unfortunately, such a situation is now encountered for sulfadoxine/pyrimethamine resistance markers in most countries in Africa where the prevalence of *dhfr*/*dhps* quadruple mutants is 50% (*24**,**25*) to 90% (*26**,**27*).

Only a few studies have investigated molecular markers of drug resistance in the context of pregnant women (*28**–**31*). The prevalence of sulfadoxine/pyrimethamine–resistant mutant parasites in pregnant women does not seem to differ greatly from the prevalence observed in the overall population. IPTp administration may induce an increase in this prevalence during pregnancy, but this increase seems limited and is not constantly observed (*28**–**31*). However, recent reports demonstrate that further drug pressure from sulfadoxine/pyrimethamine in an area of high resistance may select for a new triple mutant allele of the *dhps* gene that carries an additional mutation at codon 581 (*10*).

## Methods for Monitoring Drug Efficacy

In addition to genotyping of *Plasmodium* spp. *dhfr* and *dhps* genes, traditional methods to survey drug efficacy include in vivo and in vitro tests. The in vivo test consists of administering a curative regimen of an antimalarial drug to an infected person and following the evolution of clinical symptoms and parasite density over a few weeks. When the drug is effective, clinical signs and parasitemia levels rapidly decrease then clear, without reappearing thereafter, which is the so-called adequate clinical and parasitologic response (*32*). According to drug efficacy, parasite density only decreases or disappears but eventually reappears. To monitor drug sensitivity, children <5 years of age are the study population recommended by WHO. However, in our context, the population of choice should be pregnant women, which is consistent with the inadequacy of performing studies in children, as discussed previously.

The current policy of giving IPTp to all pregnant women generates numerous difficulties in identifying infected pregnant women and interpreting results of the tests. The prevalence of *P. falciparum* infection in pregnant women receiving IPTp is low (3% in the trial in Benin) (*33*), whereas it may reach 15%–35% in the absence of IPTp (*34**–**36*). However, one may take the opportunity to enroll women at the first antenatal care visit, when pregnancy is diagnosed, and IPTp administration has not yet started. Such a low prevalence rate will complicate identification of *P. falciparum*–infected pregnant women and will compromise enrollment of a sufficiently large number of women because this prevalence may require screening several hundred pregnant women. Conversely, treatment with sulfadoxine/pyrimethamine for a woman who has recently received (or will soon receive) a regimen of sulfadoxine/pyrimethamine as part of IPTp may increase the risk for drug hepato-toxicity and severe cutaneous side effects (*37*). In addition, such treatment with a drug that is likely to be ineffective (because parasites have persisted after IPTp with the same drug) obviously constitutes an ethical problem.

The in vitro drug assay involves culturing parasites in the presence of increasing concentrations of antimalarial drugs (in this case sulfadoxine/pyrimethamine) and determining the drug concentration that inhibits parasite maturation. For monitoring IPTp efficacy, in vitro tests are problematic because pregnant women are involved and because of the antifolate nature of the sulfadoxine/pyrimethamine. If all pregnant women are receiving IPTp with sulfadoxine/pyrimethamine, the same limitations for enrolling infected women apply, and any *P. falciparum* parasites encountered in pregnant women are likely to be resistant to sulfadoxine/pyrimethamine. Moreover, given the long half-life of sulfadoxine/pyrimethamine, many women will have residual concentrations of the drug in their blood, which may interfere with drug activity measurement. In addition, in vitro assays for sulfadoxine/pyrimethamine pose a technical challenge because they require a modified culture medium and are only partially successful compared with assays for other drugs (*38*).

## What Alternatives Can Be Proposed?

Obviously, no traditional approach is satisfactory for IPTp monitoring and one must search for alternatives. One of these alternatives may be administration of each IPTp dose to achieve a simplified in vivo efficacy test. Because each woman is given a curative dose of sulfadoxine/pyrimethamine, it should be easy to obtain a blood smear at each drug administration and to check whether parasites are cleared. The 2 curative doses of sulfadoxine/pyrimethamine are given ≈1 month apart, which corresponds to the usual follow-up period of such drug efficacy tests. A blood smear may be replaced by rapid diagnostic test or by filter paper blood spot for subsequent PCR detection of parasites. Blood spots will also enable distinguishing true therapeutic failures from reinfections by comparing the banding patterns of PCR amplicons of variable loci (such as genes for merozoite surface protein 1, merozoite surface protein 2, and glutamate-rich protein), before and after sulfadoxine/pyrimethamine administration. As mentioned earlier, the expected low prevalence of *P. falciparum* infection in this population of women receiving IPTp will explain that a high number of women under survey are likely to be required to generate useful data for public health applications.

An alternative approach involves systematic detection of placental infection at delivery by using blood smear, rapid diagnostic test, or PCR with placental blood. This method is easy to perform and would enable monitoring IPTp efficacy over several years in all centers able to diagnose malaria in an entire country. The advantage is that placental infection is a good proxy of birthweight, the major outcome in terms of public health (*39**,**40*). This approach will enable a pragmatic measure of IPTp with sulfadoxine/pyrimethamine efficacy and account for the quality of its application. Conversely, placental infection prevalence may change with time because of changes in sulfadoxine/pyrimethamine efficacy (likely to decrease) and quality of IPTp implementation (likely to increase). If the 2 variables evolve simultaneously, the resulting indicator may remain unchanged. Such an approach would also provide baseline data to assess efficacy of all preventive measures against pregnancy-associated malaria, including IPTp and use of insecticide-impregnated bed nets, and will enable assessment of these effects in a specific population. In practice, because these approaches complement each another by monitoring IPTp efficacy at different times during pregnancy, the association of these 2 approaches should be worthwhile.

## References

[1] Cot M, Deloron P. Malaria prevention strategies. Br Med Bull. 2003;67:137–48. 10.1093/bmb/ldg00314711760

[2] Desai M, ter Kuile FO, Nosten F, McGready R, Asamoa K, Brabin B, Epidemiology and burden of malaria in pregnancy. Lancet Infect Dis. 2007;7:93–104. 10.1016/S1473-3099(07)70021-X17251080

[3] World Health Organization. WHO Expert Committee on Malaria. Twentieth report. World Health Organ Tech Rep Ser. 2000;892:1–74.10892307

[4] Shulman CE, Dorman EK, Cutts F, Kawuondo K, Bulmer JN, Peshu N, Intermittent sulphadoxine-pyrimethamine to prevent severe anaemia secondary to malaria in pregnancy: a randomised placebo-controlled trial. Lancet. 1999;353:632–6. 10.1016/S0140-6736(98)07318-810030329

[5] ter Kuile FO, van Eijk AM, Filler SJ. Effect of sulfadoxine/pyrimethamine resistance on the efficacy of intermittent preventive therapy for malaria control during pregnancy: a systematic review. JAMA. 2007;297:2603–16. 10.1001/jama.297.23.260317579229

[6] East African Network for Monitoring Antimalarial Treatment (EANMAT). The efficacy of antimalarial monotherapies, sulphadoxine-pyrimethamine and amodiaquine in East Africa: implications for sub-regional policy. Trop Med Int Health. 2003;8:860–7. 10.1046/j.1360-2276.2003.01114.x14516296

[7] World Health Organization. Susceptibility of *Plasmodium falciparum* to antimalarial drugs: report on global monitoring: 1996–2004. Geneva: The Organization; 2005. WHO/HTM/MAL/2005.1103.

[8] Aubouy A, Jafari S, Huart V, Migot-Nabias F, Mayombo J, Durand R, *Dhfr* and *dhps* genotypes of *Plasmodium falciparum* isolates from Gabon correlate with in vitro activity of pyrimethamine and cycloguanil, but not with sulfadoxine/pyrimethamine treatment efficacy. J Antimicrob Chemother. 2003;52:43–9. 10.1093/jac/dkg29412805261

[9] Faucher JF, Aubouy A, Adeothy A, Doritchamou J, Cottrell G, Gourmel B, Comparison of sulfadoxine/pyrimethamine, unsupervised artemether-lumefantrine, and unsupervised artesunate-amodiaquine fixed-dose formulation for uncomplicated *Plasmodium falciparum* malaria in Benin: a randomized effectiveness noninferiority trial. J Infect Dis. 2009;200:57–65. 10.1086/59937819469703

[10] Gesase S, Gosling RD, Hashim R, Ord R, Naidoo I, Madebe R, High resistance of *Plasmodium falciparum* to sulphadoxine/pyrimethamine in northern Tanzania and the emergence of dhps resistance mutation at codon 581. PLoS ONE. 2009;4:e4569. 10.1371/journal.pone.000456919238219PMC2644264

[11] World Health Organization. Report of the technical expert group meeting on intermittent preventive treatment in pregnancy (IPTp). Geneva, 2007 July 11–13. Geneva: The Organization; 2008.

[12] Kalanda GC, Hill J, Verhoeff FH, Brabin BJ. Comparative efficacy of chloroquine and sulphadoxine–pyrimethamine in pregnant women and children: a meta-analysis. Trop Med Int Health. 2006;11:569–77. 10.1111/j.1365-3156.2006.01608.x16640608

[13] Tagbor H, Bruce J, Ord R, Randall A, Browne E, Greenwood B, Comparison of the therapeutic efficacy of chloroquine and sulphadoxine-pyrimethamine in children and pregnant women. Trop Med Int Health. 2007;12:1288–97. 10.1111/j.1365-3156.2007.01927.x17949398

[14] Mutabingwa TK, Muze K, Ord R, Briceño M, Greenwood BM, Drakeley C, Randomized trial of artesunate + amodiaquine, sulfadoxine/pyrimethamine + amodiaquine, chlorproguanal-dapsone and SP for malaria in pregnancy in Tanzania. PLoS ONE. 2009;4:e5138. 10.1371/journal.pone.000513819352498PMC2662423

[15] Briand V, Denoeud L, Massougbodji A, Cot M. Efficacy of intermittent preventive treatment versus chloroquine prophylaxis to prevent malaria during pregnancy in Benin. J Infect Dis. 2008;198:594–601. 10.1086/59011418598190

[16] Harrington WE, Mutabingwa TK, Muehlenbachs A, Sorensen B, Bolla MC, Fried M, Competitive facilitation of drug-resistant *Plasmodium falciparum* malaria parasites in pregnant women who receive preventive treatment. Proc Natl Acad Sci U S A. 2009;106:9027–32. 10.1073/pnas.090141510619451638PMC2690058

[17] Basco LK, Eldin de Pécoulas P, Wilson CM, Le Bras J, Mazabraud A. Point mutations in the dihydrofolate reductase–thymidylate synthase gene and pyrimethamine and cycloguanil resistance in *Plasmodium falciparum.* Mol Biochem Parasitol. 1995;69:135–8. 10.1016/0166-6851(94)00207-47723784

[18] Curtis J, Duraisingh MT, Trigg JK, Mbwana H, Warhurst DC, Curtis CF. Direct evidence that asparagine at position 108 of the *Plasmodium falciparum* dihydrofolate reductase is involved in resistance to antifolate drugs in Tanzania. Trans R Soc Trop Med Hyg. 1996;90:678–80. 10.1016/S0035-9203(96)90432-09015515

[19] Brooks DR, Wang P, Read M, Watkins WM, Sims PF, Hyde JE. Sequence variation of the hydroxymethyldihydropterin pyrophosphokinase: dihydropteroate synthase gene in lines of the human malaria parasite, *Plasmodium falciparum*, with differing resistance to sulfadoxine. Eur J Biochem. 1994;224:397–405. 10.1111/j.1432-1033.1994.00397.x7925353

[20] Wang P, Read M, Sims PF, Hyde JE. Sulfadoxine resistance in the human malaria parasite *Plasmodium falciparum* is determined by mutations in dihydropteroate synthetase and an additional factor associated with folate utilization. Mol Microbiol. 1997;23:979–86. 10.1046/j.1365-2958.1997.2821646.x9076734

[21] Djimdé A, Doumbo OK, Steketee RW, Plowe CV. Application of a molecular marker for surveillance of chloroquine-resistant falciparum malaria. Lancet. 2001;358:890–1. 10.1016/S0140-6736(01)06040-811567708

[22] Wellems TE, Plowe CV. Chloroquine-resistant malaria. J Infect Dis. 2001;184:770–6. 10.1086/32285811517439

[23] Laufer MK, Djimdé AA, Plowe CV. Monitoring and deterring drug-resistant malaria in the era of combination therapy. Am J Trop Med Hyg. 2007;77:160–9.18165489

[24] Tahar R, Basco LK. Molecular epidemiology of malaria in Cameroon. XXVII. Clinical and parasitological response to sulfadoxine/pyrimethamine treatment and *Plasmodium falciparum* dihydrofolate reductase and dihydropteroate synthase alleles in Cameroonian children. Acta Trop. 2007;103:81–9. 10.1016/j.actatropica.2007.04.00817640607

[25] Bonnet M, Roper C, Félix M, Coulibaly L, Kankolongo GM, Guthmann JP. Efficacy of antimalarial treatment in Guinea: in vivo study of two artemisinin combination therapies in Dabola and molecular markers of resistance to sulphadoxine-pyrimethamine in N’Zérékoré. Malar J. 2007;6:54. 10.1186/1475-2875-6-5417477865PMC1868032

[26] Ndounga M, Tahar R, Basco LK, Casimiro PN, Malonga DA, Ntoumi F. Therapeutic efficacy of sulfadoxine/pyrimethamine and the prevalence of molecular markers of resistance in under 5-year olds in Brazzaville, Congo. Trop Med Int Health. 2007;12:1164–71. 10.1111/j.1365-3156.2007.01904.x17956498

[27] Karema C, Imwong M, Fanello CI, Stepniewska K, Uwimana A, Nakeesathit S, Molecular correlates of high-level antifolate resistance in Rwandan children with *Plasmodium falciparum* malaria. Antimicrob Agents Chemother. 2010;54:477–83. 10.1128/AAC.00498-0919841150PMC2798539

[28] Mockenhaupt FP, Bedu-Addo G, Eggelte TA, Hommerich L, Holmberg V, von Oertzen C, Rapid increase in the prevalence of sulfadoxine/pyrimethamine resistance among *Plasmodium falciparum* isolated from pregnant women in Ghana. J Infect Dis. 2008;198:1545–9. 10.1086/59245518834303

[29] Bouyou-Akotet MK, Mawili-Mboumba DP, Tchantchou Tde D, Kombila M. High prevalence of sulfadoxine/pyrimethamine-resistant alleles of *Plasmodium falciparum* isolates in pregnant women at the time of introduction of intermittent preventive treatment with sulfadoxine/pyrimethamine in Gabon. J Antimicrob Chemother. 2010;65:438–41. 10.1093/jac/dkp46720053688

[30] Tinto H, Ouédraogo JB, Zongo I, van Overmeir C, van Marck E, Guiguemdé TR, Sulfadoxine/pyrimethamine efficacy and selection of *Plasmodium falciparum* DHFR mutations in Burkina Faso before its introduction as intermittent preventive treatment for pregnant women. Am J Trop Med Hyg. 2007;76:608–13.17426157

[31] Mockenhaupt FP, Eggelte TA, Böhme T, Thompson WN, Bienzle U. *Plasmodium falciparum* dihydrofolate reductase alleles and pyrimethamine use in pregnant Ghanaian women. Am J Trop Med Hyg. 2001;65:21–6.1150440210.4269/ajtmh.2001.65.21

[32] World Health Organization. Assessment and monitoring of antimalarial drug efficacy for the treatment of uncomplicated falciparum malaria. Geneva: The Organization; 2003. WHO/HTM/RBM/2003.50.

[33] Briand V, Bottero J, Noël H, Masse V, Cordel H, Guerra J, Intermittent treatment for the prevention of malaria during pregnancy in Benin: a randomized, open-label equivalence trial comparing sulfadoxine/pyrimethamine with mefloquine. J Infect Dis. 2009;200:991–1001. 10.1086/60547419656069

[34] Bouyou-Akotet MK, Nzenze-Afene S, Ngoungou EB, Kendjo E, Owono-Medang M, Lekana-Douki JB, Burden of malaria during pregnancy at the time of IPTp/SP implementation in Gabon. Am J Trop Med Hyg. 2010;82:202–9. 10.4269/ajtmh.2010.09-026720133992PMC2813157

[35] Enato EF, Mens PF, Okhamafe AO, Okpere EE, Pogoson E, Schallig HD. *Plasmodium falciparum* malaria in pregnancy: prevalence of peripheral parasitaemia, anaemia and malaria care-seeking behaviour among pregnant women attending two antenatal clinics in Edo State, Nigeria. J Obstet Gynaecol. 2009;29:301–6. 10.1080/0144361090288332019835496

[36] Ofori M, Ansah E, Agyepong I, Ofori-Adjei D, Hviid L, Akanmori B. Pregnancy-associated malaria in a rural community of Ghana. Ghana Med J. 2009;43:13–8.19652749PMC2709171

[37] Peters PJ, Thigpen MC, Parise ME, Newman RD. Safety and toxicity of sulfadoxine/pyrimethamine: implications for malaria prevention in pregnancy using intermittent preventive treatment. Drug Saf. 2007;30:481–501. 10.2165/00002018-200730060-0000317536875

[38] Basco LK. Field application of in vitro assays for the sensitivity of human malaria parasites to antimalarial drugs. Geneva: World Health Organization; 2007.

[39] Cot M, Brutus L, Pinell V, Ramaroson H, Raveloson A, Rabeson D, Malaria prevention during pregnancy in unstable transmission areas: the highlands of Madagascar. Trop Med Int Health. 2002;7:565–72. 10.1046/j.1365-3156.2002.00897.x12100438

[40] Steketee RW, Wirima JJ, Hightower AW, Slutsker L, Heymann DL, Breman JG. The effect of malaria and malaria prevention in pregnancy on offspring birthweight, prematurity, and intrauterine growth retardation in rural Malawi. Am J Trop Med Hyg. 1996;55:33–41.870203510.4269/ajtmh.1996.55.33

# Review article: Sulfadoxine/Pyrimethamine Intermittent Preventive Treatment for Malaria during Pregnancy

**DOI:** 10.3201/eid1611.101064

**Authors:** Philippe Deloron, Gwladys Bertin, Valérie Briand, Achille Massougbodji, Michel Cot

**Affiliations:** Author affiliations: Institut de Recherche pour le Développement, Paris, France (P. Deloron, G. Bertin, V. Briand, M. Cot);; Université Paris Descartes, Paris (P. Deloron, G. Bertin, V. Briand, M. Cot);; Faculté des Sciences de la Santé, Cotonou, Benin (A. Massougbodji)

## Earning CME Credit

To obtain credit, you should first read the journal article. After reading the article, you should be able to answer the following, related, multiple-choice questions. To complete the questions and earn continuing medical education (CME) credit, please go to www.medscapecme.com/journal/eid. Credit cannot be obtained for tests completed on paper, although you may use the worksheet below to keep a record of your answers. You must be a registered user on Medscape.com. If you are not registered on Medscape.com, please click on the New Users: Free Registration link on the left hand side of the website to register. Only one answer is correct for each question. Once you successfully answer all post-test questions you will be able to view and/or print your certificate. For questions regarding the content of this activity, contact the accredited provider, CME@medscape.net. For technical assistance, contact CME@webmd.net. American Medical Association’s Physician’s Recognition Award (AMA PRA) credits are accepted in the US as evidence of participation in CME activities. For further information on this award, please refer to http://www.ama-assn.org/ama/pub/category/2922.html. The AMA has determined that physicians not licensed in the US who participate in this CME activity are eligible for *AMA PRA Category 1 Credits*™. Through agreements that the AMA has made with agencies in some countries, AMA PRA credit is acceptable as evidence of participation in CME activities. If you are not licensed in the US and want to obtain an AMA PRA CME credit, please complete the questions online, print the certificate and present it to your national medical association.

## Article Title: Sulfadoxine/Pyrimethamine Intermittent Preventive Treatment for Malaria during Pregnancy

## CME Questions

**1. Which one of the following pregnant women is likely to be most vulnerable and least protected from malaria?**A. Gravida 1 para 0 (G1P0)B. G2P1C. G3P2D. G4P3**2. A 21-year-old woman residing in a stable malaria transmission area of Africa comes to the attention of the local clinic. She reports that she is feeling well and denies recent illness with or without fever.Which of the following represents current recommendations for malaria prophylaxis in this scenario?**A. Continuous chloroquineB. Intermittent sulfadoxine/pyrimethamineC. Continuous sulfadoxine/pyrimethamineD. Intermittent chloroquine**3. The clinic’s staff discusses malaria prophylaxis with the young woman who questions whether it is necessary. The clinic’s most appropriate response would be:**A. Prophylaxis is not indicated at this time because she is not currently infectedB. Prophylaxis will reduce her chances of having a low-birth-weight babyC. Prophylaxis will reduce her chances of infection during pregnancy, but is unlikely to affect the outcome of the babyD. Prophylaxis is not useful because she resides in an area with documented high resistance rates in children

## Activity Evaluation

**Table:** 

1. The activity supported the learning objectives.				
Strongly Disagree				Strongly Agree
1	2	3	4	5
2. The material was organized clearly for learning to occur.				
Strongly Disagree				Strongly Agree
1	2	3	4	5
3. The content learned from this activity will impact my practice.				
Strongly Disagree				Strongly Agree
1	2	3	4	5
4. The activity was presented objectively and free of commercial bias.				
Strongly Disagree				Strongly Agree
1	2	3	4	5
